# Teatime: epigallocatechin gallate targets fibroblast–epithelial cell crosstalk to combat lung fibrosis

**DOI:** 10.1172/JCI183970

**Published:** 2024-09-17

**Authors:** Olivier Burgy, Melanie Königshoff

**Affiliations:** 1 INSERM U1231, Faculty of Health Sciences, Université de Bourgogne, Dijon, France.; 2 Constitutive Reference Center for Rare Pulmonary Diseases–OrphaLung, University Hospital Dijon-Bourgogne, Dijon, France.; 3 Center for Lung Aging and Regeneration (CLAR), Division of Pulmonary, Allergy, Critical Care and Sleep Medicine, Department of Medicine, University of Pittsburgh, Pittsburgh, Pennsylvania, USA.; 4 Geriatric Research Education and Clinical Center (GRECC) at the VA Pittsburgh Healthcare System, Pittsburgh, Pennsylvania, USA.

## Abstract

Epigallocatechin gallate (EGCG) is a polyphenol plant metabolite abundant in tea that has demonstrated antifibrotic properties in the lung. In this issue of the *JCI*, Cohen, Brumwell, and colleagues interrogated the mechanistic action of EGCG by investigating lung biopsies of patients with mild interstitial lung disease (ILD) who had undergone EGCG treatment. EGCG targeted the WNT inhibitor SFRP2, which was enriched in fibrotic fibroblasts and acted as a TGF-β target, with paracrine effects leading to pathologic basal metaplasia of alveolar epithelial type 2 cells. This study emphasizes the epithelial-mesenchymal trophic unit as a central signaling hub in lung fibrosis. Understanding and simultaneous targeting of interlinked signaling pathways, such as TGF-β and WNT, paves the road for future treatment options for pulmonary fibrosis.

## The aberrant lung epithelium in IPF

Interstitial lung diseases (ILD) are a heterogenous group of diseases associated with progressive fibrosis (1, 2). Among them, idiopathic pulmonary fibrosis (IPF) represents a progressive fibrotic ILD of undefined etiology with a median survival of 2 to 3 years and limited therapeutic options. IPF is characterized by ill-differentiated epithelial lesions leading to distorted epithelial repair functions in the lung and activation of fibroblasts, resulting in excessive accumulation of extracellular matrix and progressive scarring. Recent single cell omics data have provided a deeper insight into pathogenic subsets of epithelial cells and fibroblasts, two cellular compartments that closely interact and form the epithelial-mesenchymal trophic unit within IPF tissue. However, the underlying mechanisms leading to the emergence of these cells, their functions, and their suitability as therapeutics targets are unknown.

**Figure 1:**
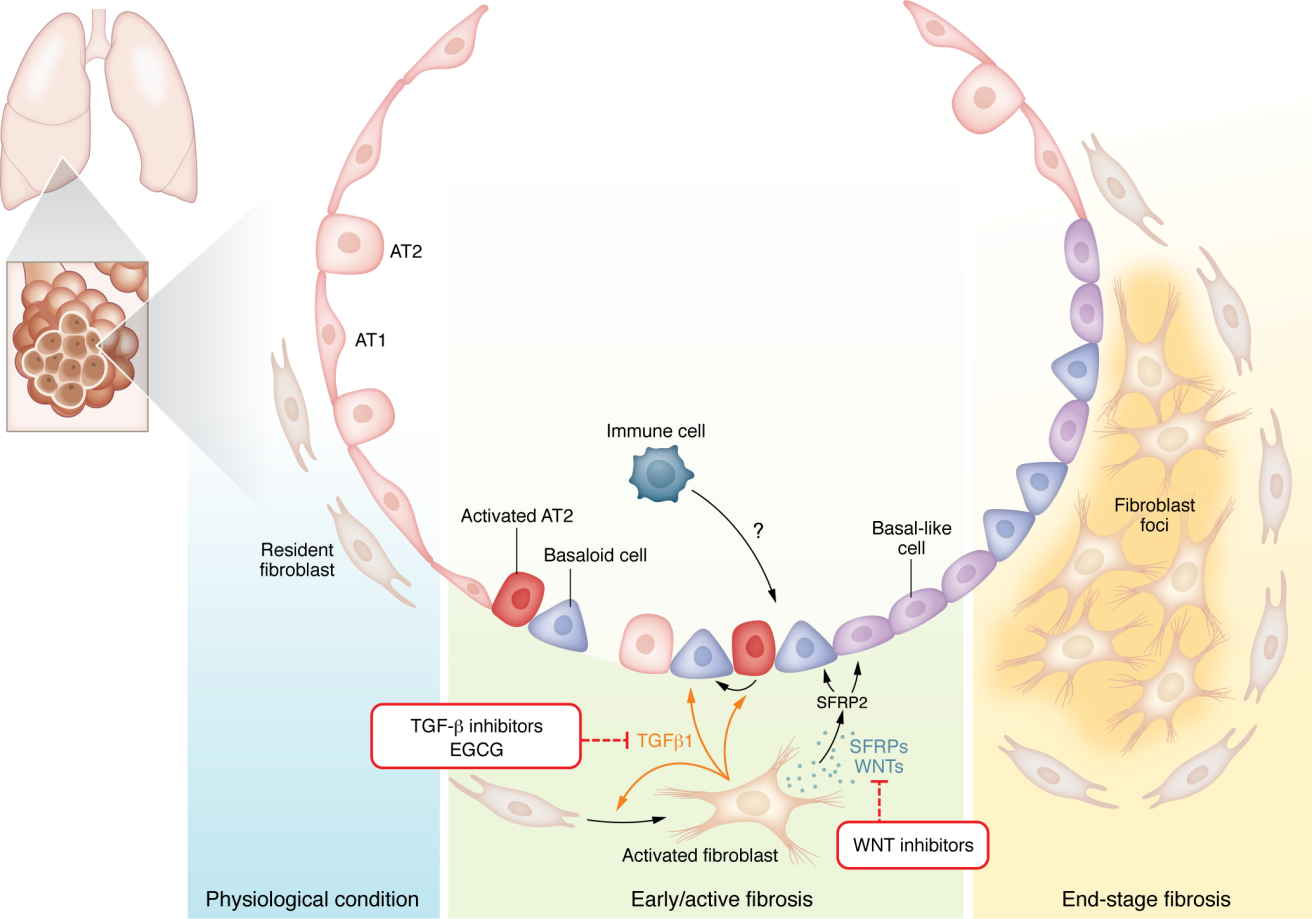
TGF-β and WNT orchestrate mesenchymal-epithelium crosstalk to drive IPF. Activated fibroblasts produce components of the WNT signaling such as SFRPs and so-called noncanonical WNT ligands in response to TGF-β. TGF-β and WNT signals act on the epithelial compartment and promote an impaired communication within the epithelial-mesenchymal trophic unit. Immune cells may also secrete factors to contribute to alveolar epithelial cell reprogramming and basal metaplasia. A consequence of this vicious central signaling hub relies on the accumulation of basaloid and basal-like cells, which will line up often in close proximity to fibroblastic foci during late-stage IPF. Potential therapeutics, such as the EGCG polyphenol, that target both TGF-β and WNT signaling hold promise to redirect mesenchymal-epithelium dialog in the organization of tissue repair.

Injury to lung epithelial cells, either by direct insults from pollutants, autoimmunity, or genetic susceptibility, is believed to be a driving force for lung fibrosis development. The distal lung is especially affected. This area includes the terminal airways and alveoli and largely comprises alveolar type 2 (AT2) cells, serving as progenitor cells, and AT1 cells, which are responsible for gas exchange. In IPF, the appearance of honeycomb cysts next to the distal subpleural airways is well described (3–7). In the IPF distal lung, intact alveolar structures with healthy AT2 cells are barely found. Rather, those areas contain Keratin 5^+^ (KRT5^+^) basal cells combined with a mix of healthy and hyperplasic alveolar epithelial cells (3–7) as well as KRT5^–^KRT17^+^ aberrant basaloid cells (8, 9). Basaloid cells have been demonstrated to contribute to fibrosis development (10). Although their origin and persistence in the fibrotic lung remain less investigated and highly debated, it is likely that these cells derive from an airway or alveolar progenitor niche. Notably, the choice of experimental models to tackle this question is crucial, as discrepancies in cell types between the human and mouse lung limits the suitability of lineage-tracing studies in mice. In particular, while aberrant basaloid cells found in human IPF share characteristics similar to those of the Krt8^+^ intermediate cells identified in mouse models (11), recent findings highlight differences in their cellular signaling pathways (12). Initial in vitro experiments using human tissue and cells suggested that AT2 cells were capable of differentiating into basal-like and basaloid cells (4, 8, 12, 13). Notably, aberrant basal-like epithelial cells are often found near activated fibroblasts and fibroblastic foci (5, 6, 12), forming the epithelial-mesenchymal trophic unit, which represents a major signaling hub in fibrosis (Figure 1).

## Developmental signaling pathways are active during fibrosis

Reactivation of developmental signaling is a major hallmark of IPF, with TGF-β being central for fibroblast activation and extracellular matrix remodeling occurring in several organs including the lung (14). Similarly, WNT signaling contributes to organ fibrosis (15, 16). WNT signaling is highly regulated by inhibitory proteins. Secreted Frizzled- related proteins (SFRPs), often described as complex WNT signal regulators, modulate the pathway by recognizing specific WNT ligands and preventing their signaling. SFRPs also bind to WNT Frizzled receptors to modify and determine signaling outcomes (15, 17, 18). The detailed signaling proteins driving fibrosis and the potential interplay between TGF-β and WNT signaling during fibrosis remain elusive.

## TGF-β/WNT signaling in the epithelial mesenchymal trophic unit

In this issue of the *JCI*, Cohen, Brumwell, and collaborators (19) aimed to provide mechanistic insight into how epigallocatechin gallate (EGCG), a polyphenol and natural plant compound most abundant in tea, mitigates lung fibrosis. The group previously demonstrated that EGCG exhibits antifibrotic effects as a fibroblast-specific, irreversible inhibitor of TGF-β receptor signaling in experimental pulmonary and cancer-associated fibrosis (20, 21). Moreover, in an exploratory study, in which EGCG was given to patients with mild ILD (that eventually advanced to IPF), EGCG decreased fibrosis markers in the lung tissue and serum samples (22). Cohen et al. further investigated this cohort and presented an in-depth analysis of single cell transcriptomics of biopsies from patients with early/mild ILD that were untreated or had undergone treatment with EGCG and compared them with IPF tissue derived from explants reflecting end-stage condition (19). Tissue access to different stages of ILD/IPF is very limited, and thus the study provides a valuable first insight into potential signaling differences across disease stages. One of the rather unexpected findings of this study is that TGF-β signaling showed highest enrichment and activity in mild ILD biopsies compared with those from end-stage disease. These findings highlight the poorly described temporal regulation of major signaling pathways, such as TGF-β, in human fibrosis, which has critical implications for therapeutic development and needs to be further dissected.

Cohen, Brumwell, and authors homed in on the EGCG-dependent reduction of TGF-β signaling within alveolar fibroblasts. Out of the most downregulated genes following EGCG, SFRP2, a soluble modulator of WNT signaling (23), was identified as a TGF-β target. Notably, SFRP2 was primarily expressed by mesenchymal cells, which express levels similar to those seen for SFRP1, a recently identified profibrotic mediator secreted mainly by fibroblasts (24). Similarly, WNT proteins themselves, primarily the ones that target noncanonical WNT signaling, such as WNT5A, are highly enriched in fibroblasts (25, 26). These studies underline the role of WNT signaling in mesenchymal cells during IPF and uncover potential regulation by TGF-β signaling. Cohen et al. show that SFRP2 inhibited WNT/β-catenin signaling, consistent with an antifibrotic role of EGCG. However, SFRP2 has been shown to also activate WNT β-catenin in cancer-associated fibroblast diseases (27). Similarly, recent studies highlight the secretion of SFRP proteins via extracellular vesicles, which potentially not only changes their signaling range, but further results in differential modulation of WNT β-catenin signaling (17, 28). Beyond their impact on WNT signaling, SFRP proteins seem to have another effect. For example, SFRP2 has been reported as a physiological enhancer of procollagen processing with potential antifibrotic effects in the heart (29). Cohen et al. started to further dissect the downstream mechanism by which SFRP2 might act and suggested that WNT receptor Frizzled 5 (FZD5) mediates SFRP2 effects on epithelial cells. Altogether, these data support a more generalizable concept in which mesenchymal cells — at least partially driven by TGF-β — act as the main paracrine regulator of WNT signaling in other lung cells, particularly epithelial cells. Further mechanistic studies are needed to delineate the cell- and signal-specific–driven effects of SFRP proteins in lung fibrosis.

Cohen, Brumwell, and authors utilized several elegant methodological approaches and advanced 3D human cell and tissue models to demonstrate that EGCG exerts its antifibrotic effects in human fibrotic tissue via TGF-β–induced SFRP2. Importantly, they expanded upon a previously published human AT2 cell–based organoid model in which AT2 cells differentiate into basal-like cells. These findings demonstrate that SFRP2 promotes basal cell markers and features in healthy AT2 cells in vitro. These data were further corroborated in human tissue–derived precision cut lung slices (PCLSs) ex vivo, which allowed investigation of the naive 3D distal lung structure lung’s native environment. Cotreatment with TGF-β and SFRP2 led to induction of the basal cell marker KRT5, which was not expressed at baseline. Thus, these data add to the growing evidence that AT2 cells in their naive environment are indeed able to gain a basal-like phenotype, further highlighting the distal epithelium as a primary target side for IPF.

While SFRP2 emerges as a central mediator and potential therapeutic target downstream of EGCG, it is likely that EGCG exerts additional effects within the fibrotic lung, which remain to be investigated. The authors focused primarily on mesenchymal subsets, while the (direct or indirect) influences of EGCG on other cell types, such as epithelial or immune cells, require further exploration. Of note, EGCG affected inflammatory pathways, further underpinning the necessity for a more in-depth analysis.

Overall, the unique strengths of the Cohen et al. study lie in its initial approach: subjecting human lung tissue biopsies from patients under a EGCG regimen to single-cell omics analysis, thereby providing a clinically relevant proof demonstrating the mechanism of the drug in situ in a real-world setting. The study further corroborates antifibrotic effects of EGCG and highlights the emerging role of fibroblast-derived SFRP proteins as an early paracrine signaling mediator contributing to the emergence and potential maintenance of aberrant epithelial cells in IPF.

## Version 1 09/17/2024

Electronic publication
